# Depression and social isolation during the COVID-19 pandemic in a student population: the effects of establishing and relaxing social restrictions

**DOI:** 10.3389/fpsyt.2023.1200643

**Published:** 2023-08-07

**Authors:** Rainer Matthias Holm-Hadulla, Hannes Wendler, Gabriella Baracsi, Timo Storck, Andreas Möltner, Sabine C. Herpertz

**Affiliations:** ^1^Department of General Psychiatry, University Hospital Heidelberg, Heidelberg, Germany; ^2^Department of Psychiatry and Mental Health East, Faculty of Medicine, University of Chile, Santiago, Chile; ^3^Department of Philosophy, University of Cologne, Cologne, Germany; ^4^Psychologische Hochschule Berlin, Berlin, Germany; ^5^Dean’s Office of the Medical Faculty of Heidelberg University, Heidelberg, Germany

**Keywords:** depression, anxiety, stress, well-being, social isolation, loneliness, COVID-19, students

## Abstract

**Introduction:**

In a quasi-naturalistic study design, we evaluate the change in psychopathological syndromes and general well-being after the alleviation of social restrictions. The aim of this study was to investigate the specific relationship between social isolation and depressive syndromes.

**Methods:**

At two timepoints, the first during maximal social restrictions, the second after social restrictions had widely ended for 9 months, depressive and other syndromes were measured in an online survey addressing the total cohort of students registered at Heidelberg University, Germany via e-mail (*n* = 27,162). The complete Patient Health Questionnaire (PHQ) was used with nine items for depressive syndromes. In addition, well-being was measured by the Well-Being Index WHO-5. In the quantitative and qualitative part of the study psychopathological syndromes and well-being were related to social isolation and feelings of loneliness.

**Results:**

After 1.5 years of pandemic-related social restrictions, “major” depressive syndromes were reported by 40.16% of the respondents to the PHQ in a sample of 2,318 university students. 72.52% showed a severely reduced Well-Being-Index. Nine months after the end of social restrictions, “major” depressive syndromes were reported by 28.50% of the participants. Well-being improved after the alleviation of social restrictions, as well: 53.96% showed a Well-Being Index of below 50 vs. 72.52% in the first study. The quantitative and qualitative analysis of the free texts of the respondents suggest that a significant amount of depressive syndromes and reduced well-being are related to social isolation and loneliness. While in the times of the pandemic restrictions the participants mostly reported “loneliness and social isolation” (24.2%) as their main problem, only 7.7% described these as their main problem after social restrictions had been loosened for 9 months. The qualitative analysis hints that at t2 participants were more likely to mention possible ways to actively deal with loneliness than at t1, which might be interpreted along the lines of the decrease in depressive syndromes.

**Discussion:**

Keeping the self-selection bias in mind our study results suggest that one third of “major” depressive syndromes and one quarter of severely reduced well-being accompany social restrictions or are even caused by them, with loneliness being an important factor. These results should be taken into account by health policies when coping with future pandemics.

## Introduction

The COVID-19 pandemic has severely impacted physical and mental health. During the pandemic, a significant increase in somatic symptoms (such as fatigue) and mental health concerns such as dysfunctional coping strategies (i.e., COVID-19 anxiety syndrome) or allostatic overload were observed (1–3). Regarding the decline of mental health, depressive symptoms featured among the most prominent (4). According to the bio-psycho-social framework of depression there is a complex interaction of biological and psychological factors with social influences (1–3). Research on creativity and depression shows how dealing with loneliness accounts for human cultural productivity as well as its impasses. Depression is one of the mental and neurological manifestations of COVID-19.Here, we focus on the influence of social isolation on the prevalence of depressive syndromes. During the COVID-19 pandemic, an increase in mental disorders was registered worldwide (5–7). Studies from all over the globe reported increased levels of loneliness related symptoms, such as depression, anxiety, stress, and Covid-fear (8–12). Higher levels of psychopathology, namely depression but also anxiety and COVID-fear, may at least partially result from social isolation (8). During the pandemic-related social restrictions a study in China with 746,217 students showed prevalence rates of an acute stress reaction in 34,9%, of depressive syndromes in 21,1% and of anxiety syndromes in 11,0% (6). In a US study with 45,000 participants, 35% of undergraduate and 32% of graduate students were screened positive for “major depressive disorders” during pandemic-related social restrictions (7). A study in Bangladesh found that almost 69.3% of college and university students partaking experienced event-specific stress during the COVID-19 pandemic, with 46.9% being depressed and 33.3% suffering from anxiety (10). Overall, ample evidence suggests that social restrictions in the context of the pandemic lead to feelings of loneliness and dramatically impact mental health (13–17). During the COVID-19 pandemic, public measures related to lockdowns such as physical distancing, work disruptions, school closings, and mobility restrictions profoundly changed social life and daily routines (18–25). The reduction of social contacts, with a consequent increase in social isolation and feelings of loneliness, was associated with increased prevalence of depression, anxiety and suicidal behavior (14, 26).

Loneliness is generally seen as a risk factor for many mental disorders such as depression, anxiety, and stress (22, 27–29). A United States study found that 43% of respondents exhibited elevated levels of loneliness, which was associated with depression and suicidal ideation. Especially for women, younger and less educated persons, social isolation due to pandemic-related restrictions led to depression and feelings of loneliness (15, 30–32). Stress has been shown to predict depression directly, whereas COVID-fear connection to depression is mediated through anxiety (33, 34). Covid-fear is significant for other mental health issues as well, such as obsessive–compulsive disorder and substance use (35, 36).

## Research hypothesis

In a quasi-naturalistic study design, we evaluate the change in psychopathological syndromes and general well-being after the alleviation of social restrictions. The aim of this study was to explicate the specific relationship between social isolation and depressive syndromes. This could be achieved by investigating whether and to what extent depressive and other psychopathological syndromes like anxiety, somatoform, alcohol and bulimic syndromes decreased after pandemic-related social restrictions had been loosened or reversed for 9 months. Our main hypothesis was that depressive syndromes decreased to a larger extent compared to other syndromes because results of a pre-study conducted while public, professional and social life were restricted showed that depression was more often attributed to loneliness due to social restrictions during the COVID-19 pandemic than other syndromes were (5).

## Methods

After approval by the Ethics Committee of the University Hospital and the Data Protection Officer of Heidelberg University, the totality of all students of Heidelberg University (*n* = 27,162) were asked per e-mail to participate in an online survey. The survey was completely anonymous. The first survey took place between May 26th, 2021 and June 11th, 2021 via the Limesurvey” platform while the aforementioned social restrictions had been set up for one and a half years. Data of this survey have been published in 2021 (5). The second survey took place from May 25th, 2022 to June 10th, 2022 after the social restrictions had been relaxed for 9 months, and, thus, exactly 1 year after the 1st survey. In order to protect the security of the sensitive data, also to get reliable answers due to trust, the email addresses of the respondents were not stored, so all students at Heidelberg University were asked to complete the questionnaire in both years. In addition, a sub-sample was formed consisting of those respondents who indicated that they had participated in each year. The financial background of the students is stable; there is no university fee, also the students have an opportunity to get financial support during their studies, such as the education advancement grants (Ausbildungsförderung, BAföG). Demographic variables collected included age, gender, and field of academic study. These categories were the same in each year.

## Investigative tools

Mental health symptoms were assessed with the German version of the Patient Health Questionnaire (PHQ-D) (37) containing nine items for depressive syndromes (PHQ-9) and seven items for anxiety syndromes (PHQ-7) (27). In international comparison, the PHQ is the most frequently used screening instrument to assess depression and anxiety as well as somatoform (13 items) and alcohol syndromes (six items, five of which were used). Therefore, it is not a diagnostic test, but rather an exploration of signs of the level of depression and anxiety syndromes. Especially the PHQ subtests for depressive and anxiety and somatoform syndromes show a high reliability: internal consistency is Cronbach s = 0.88 for the depression module and the anxiety module, and α = 0.79 for the somatoform module (30). Test–retest reliabilities are *r* = 0.83 and *r* = 0.84, respectively, and the reliabilities for self- and external evaluation are also *r* = 0.83 and *r* = 0.84 (38).

To measure the change of well-being between the two -surveys we used the German version of the WHO-5 Well-Being Index (39). The WHO-5 is used as a screening instrument to measure subjective well-being (38) and allows international comparisons. It has a high internal consistency of Cronbach s = 0.88.

Differences in dichotomous variables between the two surveys were tested by Fisher’s exact test (hybrid form according to Mehta and Patel (22)), differences of ordered categorical variables by Jonckheere-Terpstra tests and of categorical variables by *χ*^2^-tests.

The entire quantitative data analysis was carried out using R Version 4.1.0. The R packages “psych,” “clinfun” and “crosstable” were used for the calculation of descriptive parameters and statistical tests. For PHQ-D and WHO-5, comparisons were made of the descriptive data with corresponding norm values for students and other populations.

Other measures deployed in the study were Sense of Coherence Scale (SOC), Brief COPE, Social Support Inventory (ESSI-D), Interpersonal Reactivity Index (IRI), and General Self-Efficacy Scale (SGSE). This comprehensive set of tests took participants about 60 min in average to complete.

## Qualitative analysis

Participants also were asked to freely comment on the main complaints concerning the pandemic and the related restrictions. In addition to make proposals on how to improve their situation. In a first survey, 2,103 persons responded, in the follow-up, 581 did. For qualitative analysis we conducted thematic analysis. Thematic analysis is a method of analyzing qualitative data in which a data set is searched to identify, analyze and report recurring patterns (40). The analyzing process is conducted through multiple steps: from getting to know the entire dataset, to creating a definition and narrative description of each theme and to the final analysis and description of the results (40, 41). The method lends itself to identifying, analyzing and presenting themes or patterns in a sample, based on the analysis of categories. Through its theoretical freedom, thematic analysis offers a flexible and explorative research tool that can potentially provide a rich and detailed, yet complex, account of the data. Thematic analysis was developed to look for common or shared meanings and not to understand single individuals’ unique experiences, so it is an efficient method to use for large samples (41).

## Results

In the first survey, 2.135 students completed the extensive questionnaire and were included in the analysis. The whole response rates of 8.8% were much higher than those of the regular surveys of the German Student Union (Deutsches Studierendenwerk), where response rates of between 2 and 3% are achieved (42). In the second survey, 682 students completed the demographic inquiry as well as the WHO-5, 599 also completed the PHQ. The difference in response rate will be discussed later; regarding the mere fact of eased restrictions in terms of social isolation as well as the hypothesis that more persons more severely strained might have a greater urge to have these strains recognized, this is not surprising.

There are no significant differences between the respondents of the first and second survey in respect to gender, age distribution, and field of academic studies (see Table 1). Given the large n, even small differences tend to show statistical significance which, due to their marginal importance, were disregarded in the present analysis.

**Table 1:** Participants’ age, gender and field of academic studies.

Variable		2021		2022		*p*
		*N*	%	*N*	%	
Age	Under 21	662	27.61	173	24.68	0.1790^1^
	21–23	941	39.24	274	39.09	
	24–25	392	16.35	143	20.40	
	26–27	161	6.71	45	6.42	
	Over 27	242	10.09	66	9.42	
Gender	Male	780	32.53	254	36.23	0.0210^2^
	Female	1,578	65.80	416	59.34	
Field of studies	Humanities	562	23.44	175	24.96	0.0423^3^
	Law	242	10.09	52	7.42	
	Medicine	372	15.51	89	12.70	
	Mathematics/natural sciences	729	30.40	246	35.09	
	Psychology/social sciences	305	12.72	87	12.41	
	Others	188	7.84	52	7.42	

Tests of differences between surveys: ^1^ Jonckhhere-Terpsta test, ^2^Fisher’s Exact Test, ^3^*χ*^2^-Test.

The most prominent finding of the present study is that “major depressive syndromes” decreased significantly from 40.16 to 28.50%. Also “other depressive syndromes” decreased significantly from 16.92 to 11.33 (see Table 2). The average depression score of the PHQ improved from 11.61 (SD: 6.09) in the first survey, to 10.22 (SD: 6.25) in the second survey (Student’s *t*-test: *p* < 0.001).

**Table 2:** Frequencies of syndromes in survey 2021 and 2022 according to the PHQ.

Syndrome	Sex	2021		2022		*p* ^1^
		*N*	%	*N*	%	
Major depressive syndrome		2,139	40.16	600	28.50	<0.001
	f	1,421	41.87	360	27.78	<0.001
	m	682	35.78	217	29.03	0.071
Other depressive syndromes		2,139	16.92	600	11.33	<0.001
	f	1,421	15.97	360	10.00	0.004
	m	682	19.06	217	12.44	0.024
Panic and anxiety syndromes		2,137	19.98	599	17.53	0.199
	f	1,419	23.82	360	18.89	0.049
	m	682	12.02	217	13.82	0.480
Stress ≥10		2,133	17.16	599	16.36	0.667
	f	1,419	19.73	360	17.50	0.370
	m	678	11.95	217	14.29	0.409
Somatoform syndrome		2,139	25.39	600	21.17	0.036
	f	1,420	33.10	360	27.78	0.058
	m	683	9.37	217	9.22	1.000
Bulimia/binge syndrome		2,138	8.33	599	9.02	0.618
	f	1,420	8.59	360	10.56	0.256
	m	682	7.77	217	6.91	0.769
Alcohol syndromes		2,135	9.88	599	9.52	0.876
	f	1,418	8.25	360	8.61	0.831
	m	681	13.07	217	11.98	0.727
WHO-5; 100 < 50		2,358	72.52	682	53.96	<0.001
	f	1,552	73.78	407	52.09	<0.001
	m	767	69.62	247	53.85	<0.001

^1^Tests of differences between surveys: Fisher’s Exact Test.

Somatoform syndromes differed slightly but not significantly between the two surveys (25.39% vs. 21.17%). Generalized anxiety and panic syndromes did not differ significantly either (19.98% vs. 17.53%). Also, general stress syndromes were nearly the same (17.16% vs. 16.36%) as were signs of abuse of alcohol or addiction (9.88% vs. 9.52%) and bulimia and binge eating syndromes (8.33% vs. 9.02; see Table 2).

Also, in the analysis of continuously divided depressive syndromes, there were significant and clinically relevant differences between the two surveys. A score of less than 5, indicating “no or minimal” impairment, showed 12.2% of the respondents in the first survey vs. 18.3% in the second one. A score of 5–10, indicating “mild depressive” impairment, reported 28.7% vs. 33.7% of the respondents. A score of 10–14, which is seen as an indication of “moderate depressive” impairment, was shown by 27.3% vs. 24.2%. A score of 15–19 indicating “moderately to severe” symptoms was found in 20.0% vs. 14.2%. and a score of 20–27 indicating “severe depressive” symptoms had 11.9% vs. 9.7% of the respondents. This means while 31.9% of the students in the first survey showed indications of “moderately to severe” or “severe depression” only 23.9% showed indications of “moderately severe” or “severe depression” in the second survey.

The high extent of depressive syndromes and their change after 9 months of relaxed social restrictions corresponds to the finding that well-being measured by the WHO-5 also improved in a statistically significant and clinically relevant amount from 37.56 (SD 21, 27) to 47.17 (SD 21.99) (26, 43). While 72.52% of the respondents showed a severely impaired well-being in the first survey, 53.96% showed a WHO-5 Well-Being of lower than 50 in the second survey (see Table 2).

With regard to gender, it is noticeable that “major” depressive syndromes were present in 41.87% vs. 27.78 of women (*n* = 1,419) and in 35.78% vs. 29.03% of men (*n* = 682), when comparing the two surveys. Somatoform syndromes were found in 33,10% vs. 27.78% of women and 9.37% vs. 9.22% of men. Panic and anxiety syndromes were present in 23.82% vs. 18.89% of women and 12.0% vs. 13.8% of men (see Table 2).

### Results of qualitative analyzes

In this mixed-designed study, there was a qualitative part in the questionnaire which was used to get a deeper understanding of the students and the situation they experienced. Using the PHQ-14 questionnaire’s closing question: *“What are your main complaints?”* students showed remarkable differences between the timepoint after 1.5 years of severe social restrictions and a timepoint after 9 months of loosened or no social restrictions.

Nine categories could be discerned within the free-form answer texts and were connected to anchoring examples in the text as well as rules for further coding. Among these nine categories, “social isolation and loneliness” was the one with most single codings, with 24.2% of respondents claiming to suffer from it, while only 7.7% of the respondents in the 2022 post-survey did. Typical comments regarding the most urgent strains at the time were as follows: *“That you spend so much time alone. Usually, a friend or family member would notice when you are not well. Now you sit at home alone in such situations, you do not call anyone because you know that your friends have enough problems themselves at the moment and therefore you do not get the mental support that you would need in some moments.”* Although many respondents commented on it, the category “study related stress” was less frequent than “social isolation and loneliness.” Typical comments were: *“Studying; worrying about taking the exam; worrying about patients dropping out during treatment; worrying about whether you’ll pass the course..”*

In contrast, the main complaints in 2022 were “study related stress” increasing to 26.9% of the participants from 15.7% in the pre-survey. Typical answers were as follows: *“Feeling like I’m not doing enough for my studies and that others are doing everything better than me.” “Anxiety attacks before exams.” “Stress about university and work..”* Notably the theme of “loneliness” with 7.7% was considerably less frequent in 2022 compared to 24.2% in 2021. Notably, in some students the former pandemic-related social restrictions continued to have an impact. Typical reports sounded like: *“I lack friendships at university because I started to take courses during the pandemic and since then I did not have the chance to find the contacts that I lacked at the beginning of my studies.”*

In summary, in 2021 most students reported on suffering under “social isolation and loneliness,” whereas in 2022 most suffered under “study related stress.” In relation to the results from the quantitative part of the study, these findings appear to be most relevant. The details of the qualitative analysis will be published in a separate article.

## Discussion

This significant lower response rate (2.5%) to the extensive questionnaire might be due to a self-selection bias (28). This response rate is similar to with comparable studies in populations of German students (39). However, one could argue that filling out an online survey on social isolation in the times of pandemic restrictions might itself be influenced by the study’s subject matter. Therefore, the decrease in response rate might be due to less burden of disease and more social activities.

Focusing on the impact of social restrictions during the COVID-19 pandemic, this study compared the frequency of psychopathological syndromes and impairment in well-being in a large student population at a timepoint of high social restrictions versus a timepoint 1 year later when social restrictions had been widely relaxed for 9 months. Data shows that social restrictions are related to an increase of depressive syndromes and a decrease of well-being while relaxation of severe social restrictions is related to a reduction of depressive syndromes and improved well-being as Wasserman et al. (26) proposed. After 9 months in which interpersonal interactions were possible again on campus and in social life elsewhere “major” depressive syndromes among students decreased from 40.16 to 28.50%. This, however, still exceeds the rate of 22.7% of persons burdened by depressive syndromes in a comparable prepandemic study of students (29).

While the amount of other syndromes like those related to anxiety, somatoform, alcohol, bulimia and binge eating syndromes did not change significantly, our study shows that about one third of the depressive syndromes improved after the relaxation of social restrictions. This underlines the supposition of Wasserman et al. (26) that depressive syndrome may be triggered or even caused by social restrictions leading to loneliness. This is also consistent with findings reporting an association between mental health and a variety of lockdown measures, such as school closings, workplace-disruptions or transport restrictions (44–47). To borrow the phrase of Killgore and colleagues (17), loneliness must be considered *a signature mental health concern in the era of COVID-19* (13, 17).

The impairment of well-being is statistically significant and clinically relevant in times of long-lasting social restrictions which, in case of the time in which the first survey was undertaken, had been lasted for approximately 14 months (28, 42). After relaxation of the restrictions well-being is improved. While during the social isolation nearly three quarters of the respondents showed low well-being, it was about two quarters after the relaxation of social restrictions. Notably, the average WHO-5 Well-Being Index score of 47.2 remains to be considerably lower than in the pre-pandemic studies in Germany, for example, 65.0 in 2016 (29, 30) and 57.0 in 2020 (31). This is not surprising since pandemic-related restrictions might have had an impact on some students´ long-term social life.

Even though an in-depth analysis of the qualitative data is beyond the scope of this paper (especially focusing on overarching themes across different self-reports of participants which might further show the relations between feeling burdened by social isolation, online-only university courses and dissatisfaction with institutional support), results of the qualitative analysis underline that social restrictions leading to loneliness is a relevant factor in the pathogenesis of depressive syndromes and reduced well-being. This result convenes with the study of Wassermann et al. (26) which showed that decreased opportunities to contact people outside home have a negative impact on mental health. While loneliness and suffering under the social restrictions were the main complaints in our pre-survey, everyday stress with academic studies was the main complaint in the post-survey. Consequently, participants in the first survey most frequently stated that a loosening of social restrictions would improve their situation. In the second survey the most frequent proposals of the students focused on the reduction of exam stress and problems to academic studies. These self-reports have to be interpreted in light of the findings suggesting that the duration of loneliness is more strongly correlated with mental health symptoms than its intensity (15). We suppose that testing this hypothesis is an interesting venue for further research.

Also, qualitative analysis hints that in 2021 more participants were likely to report *complaints* (regarding state and university decisions in handling the pandemic) while in 2022 they were more likely to state their *claims* and wishes. This can be interpreted as a shift from a “depressive” and passive stance in dealing with burdens in 2021 to a more active way of self-management in 2022. After all, while social restrictions took a huge part in an increase of depressive syndromes, the conclusion that easing restrictions will in turn lead to health and well-being is questionable. Qualitative data suggest, however, that in 2022 participants reported a “healthier” (in the sense of increased self-efficacy) way of managing problems. Easing restrictions, then, would probably not directly lead to well-being but to empowering participants to actively change conditions in which they do not feel well (e.g., seeking social contact) and thus no longer meet depressive syndrome criteria.

Compared to the 21.1% of depressive syndromes in the 2020 comprehensive study during high social restrictions in China by Ma et al. (6) which used similar measures as our study, our second survey presents with 28,50% of “major” depressive syndromes even higher scores at a timepoint after social restriction measures had stopped. However, in the study of Ma et al. “acute stress syndromes” were with 34.9% much more frequent than in our studies (17.16% in 2021 and 16.36% in 2022). The percentages of “major” depressive syndromes in our post-survey are distinctly lower than those of a comprehensive study from the United States with 30,725 undergraduate students and 15.346 graduate and professional students conducted in May–July 2020 at 9 public research universities by Chirikov et al. (48). This study shows that the prevalence of depressive syndromes among students was twice as high in 2020 as in 2019, with 35% of undergraduate and 32% of graduate students showing “evidence” of “major depressive disorders.” In the year before the pandemic Chirikov et al. (48) showed, that only 15% of students presented with indications of “major depression.” The main shortcoming of the study by Chirikov et al. (48) is, that it is based on the PHQ-2, which consists of only 2 items. Other obligatory symptoms for the diagnosis of major depression in the sense of the International Classification of Disease (ICD-10) and the International Diagnostic Statistical Manual (DSM-5-R), such as reduced activities and somatic syndrome, are thus not assessed. In this respect, the term “major depression” is not appropriate from a clinical-psychiatric perspective. The application of the PHQ-9 is more advanced because it takes into account the breadth of depressive symptoms with lack of activities, joylessness and somatic syndrome in addition to the mood disorder.

## Conclusion

Keeping the self-selection bias in mind (49), our results strongly suggest that relaxing social restrictions and alleviating loneliness improves well-being and depression of many students in a significant and clinically relevant way. Future lockdown policies should take these results into account, e.g., by controlled facilitation of personal encounters in the form of face-to-face teaching and enabling of social contacts in seminars, refectories, libraries, sports facilities, cultural events, etc. This is also relevant for dealing with future pandemic outbreaks or other crises. The impact of social lockdowns is severe for many people. In sum, loneliness must be considered a “signature mental health concern in the era of COVID-19” (13). Thus, preventing loneliness and maintaining ways to actively manage crises amounts to a major public health concern in future lockdown policies. The current major focus is on providing the necessary treatment to the many young people who suffer from depressive syndromes related to social restrictions and loneliness. Our findings underscore the importance of improving social contact to reduce negative impact of the COVID-19 pandemic. Clinicians are thus encouraged to focus on interventions that strengthen and enable social interactions.

Under pandemic conditions, we should support not only students but also other populations and especially persons lacking social networks (45) since they are more vulnerable to mental illness in times of pandemic crises (31). Also, one should bear in mind the preventive role social interaction in quasi-institutional contexts such as universities, schools or sports clubs and others play. Those can serve as a “quasi-vaccination” against mental burdens stemming from loneliness. Special resources should be allocated to maintain a minimum of social life and self-management even in times of crises. Personal contacts are indispensable for well-being, mental health, and social relatedness (48, 50). The vast majority of students long for opportunities to develop socially and want to contribute to professional, scientific, and humanistic progress in line with the guiding principle of the World Health Organization (WHO): more (socially) active people for a healthier world (44).

## Limitations

The following limitations have to be considered: (1) The results presented here, though making a strong case for keeping in mind the impact of (restricted) social life on mental health, can only highlight influencing factors. There might be other factors contributing to feeling less burdened at the timepoint of our second survey, most notably having more knowledge about COVID-19 itself or access to vaccination (which might turn out to influence both relaxing social restriction by political institutions and a reduction of mental health problems in individuals). Surely, there has been a complex interplay of personal, societal, medical, and political development during the pandemic’s first 2 years. (1) Notably, the response rate to the extensive questionnaire in our first survey which was much higher than in the regular surveys of the German Student Union could not be preserved in the second survey reported here. (2) We cannot exclude a self-selection bias, neither in the first nor in the second survey. Those students particularly affected by pandemic-related restrictions may have responded more frequently in the first and in the second study. Less concern among students at the time of the second survey, however, may reason less participation in the follow-up and, thus, may also be due to self-selection. This self-selection bias, which also applies to comparable studies from Germany, United States, and China does not detract from the central finding that severe social restrictions and resulting loneliness are related with an increase of depressive syndromes and a decrease of well-being. (3) Future studies on the sequelae of pandemics or other societal crises on mental health issues should include quantitative measurements of loneliness and isolation in addition to the qualitative method we applied in this study. Also, extended qualitative measures, e.g., retrospective interviews to address long-term negative effects of social restrictions on social life and well-being, are favorable. (4) The anonymous survey design allows only for group comparisons. Further limitations are that we used only self-reports and that the PHQ and other of the employed measures have not been validated specifically in the student population. The fact that no incentives were given to the students may have reduced the response rates, but at the same time makes it more probable that only serious and reliable answers were given.

## Data availability statement

The raw data supporting the conclusions of this article will be made available by the authors, without undue reservation.

## Ethics statement

The studies involving human participants were reviewed and approved by Ethics Committee of the University Hospital. The patients/participants provided their written informed consent to participate in this study.

## Author contributions

RH-H initiated and designed the study and wrote the 1st version of the paper. HW, GB, TS, AM, and SH participated in the data collection and analysis of the data. All authors discussed jointly the results and wrote together the final version of the manuscript.

## Funding

For the publication fee we acknowledge financial support by Deutsche Forschungsgemeinschaft within the funding programme “Open Access Publikationskosten” as well as by Heidelberg University.

## Conflict of interest

The authors declare that the research was conducted in the absence of any commercial or financial relationships that could be construed as a potential conflict of interest.

## Publisher’s note

All claims expressed in this article are solely those of the authors and do not necessarily represent those of their affiliated organizations, or those of the publisher, the editors and the reviewers. Any product that may be evaluated in this article, or claim that may be made by its manufacturer, is not guaranteed or endorsed by the publisher.
